# Total Harmonic Distortion of a Piezoelectric MEMS Loudspeaker in an IEC 60318-4 Coupler Estimation Using Static Measurements and a Nonlinear State Space Model

**DOI:** 10.3390/mi12121437

**Published:** 2021-11-24

**Authors:** Romain Liechti, Stéphane Durand, Thierry Hilt, Fabrice Casset, Christophe Poulain, Gwenaël Le Rhun, Franklin Pavageau, Hugo Kuentz, Mikaël Colin

**Affiliations:** 1 University Grenoble Alpes, CEA, Leti, F-38000 Grenoble, France; thierry.hilt@cea.fr (T.H.); fabrice.casset@cea.fr (F.C.); christophe.poulain@cea.fr (C.P.); gwenael.le-rhun@cea.fr (G.L.R.); franklin.pavageau@cea.fr (F.P.); hugo.kuentz@cea.fr (H.K.); mikael.colin@cea.fr (M.C.); 2 Laboratoire d’Acoustique de l’Université du Mans, LAUM-UMR 6613 CNRS, Le Mans Université, F-72085 Le Mans, France; stephane.durand@univ-lemans.fr

**Keywords:** MEMS, piezoelectric, loudspeaker, total harmonic distortion, state space, IEC 711, thin film, PZT

## Abstract

We propose a method to evaluate the Total Harmonic Distortion generated by a cantilever-based PZT loudspeaker inside an IEC 60318-4 coupler. The model is validated using experimental data of a commercial loudspeaker. Using the time domain equations of the equivalent electrical circuit of the loudspeaker inside the coupler and a state space formulation, the acoustic pressure response is calculated and compared to the measurement of the manufacturer. Next, the stiffness, transduction and capacitance nonlinear functions are evaluated with a Double-Beam Laser Interferometer (DBLI) and a nanoindenter on test devices and on the commercial loudspeaker. By introducing the nonlinear functions into the model as amplitude-dependent parameters, the THD generated by the loudspeaker is calculated and compared to the value provided by the manufacturer. The good agreement between the measurement and the simulation could allow for a rather quick simulation of the performance of similarly designed loudspeakers at the early stage of the design, by only estimating the static linearity of the main nonlinearity sources.

## 1. Introduction

Loudspeakers are used to converting an electrical signal into sound waves, as accurately as possible, with a sufficiently high sound pressure level. This is usually achieved with a piston-like membrane, behaving as a first-order oscillating system, actuated by an electromechanical transducer. Driven by the forever growing industry of portable and connected devices, research was carried out in order to make the loudspeaker compatible with microfabrication processes, in order to reduce the manufacturing tolerances, the cost and the thickness of the devices. An interesting performance was demonstrated with Micro Electromechanical Systems (MEMS) electrodynamic loudspeakers [1,2] and pieozoelectric loudspeakers [3,4,5,6,7]. Nevertheless, the achieved performance is not competitive compared to non-MEMS loudspeaker. However, as in-ear sound generation requires less mechanical displacement, MEMS loudspeakers have shown interesting results in terms of frequency response and sound pressure level when evaluated in couplers [8,9,10]. A few MEMS loudspeakers for in-ear applications have demonstrated to fulfill the industry required performances, with electrostatic [8] and piezoelectric transduction [9,10].

The second most important characteristic of a loudspeaker after its frequency response is its linearity. This characteristic is often evaluated using the Total Harmonic Distortion (THD). The THD is the ratio of the power of all harmonics over the total power. Studies have shown that depending on the order of the harmonics, the human ear can be sensitive to a THD as low as 0.1% [11,12,13,14]. Electrostatic transduction is inherently nonlinear, and the reachable displacement is not sufficient to advantageously replace non-MEMS electrodynamic loudspeakers. The most promising transduction is the piezoelectric transduction and more specifically the one using PZT (lead zirconate titanate) actuators, providing a high transduction factor for low actuation voltages in the case of thin films. Unfortunately, thin-film PZT shows a ferroelectric and electrostrictive behavior, creating a wide variety of nonlinearities [15,16,17,18].

To model the nonlinearities of classical electrodynamic loudspeakers, state space models, port-Hamiltonian systems, Hammerstein models and power series have been extensively used [19,20,21,22,23,24,25]. State space models are the most commonly used models to simulate nonlinear effects in electrodynamic loudspeaker and have shown accurate results. However, for piezoelectric transduction, such a model has not been reported yet. Recently, a model for the simulation of nonlinear effects in coupler for electrostatic transduction was reported [26]. Accurately, modeling the nonlinear behavior of loudspeakers from static nonlinear measurements of the transduction transfer function could be an efficient way for estimating the total harmonic distortion, without using expensive prototypes. In addition, an accurate model of the nonlinear behavior of the loudspeaker could help developing methods to actively reduce the nonlinearities, as it has been extensively performed with electrodynamic non-MEMS loudspeakers [27]. In addition, accurately simulating the nonlinearities generated by a loudspeaker could be used to simulate the subjective audio quality of the loudspeaker in the early phases of the design, using models of the perceptual components of the loudspeaker [28,29,30,31]. The aim of this study is to provide such a model to evaluate the distortion generated by a cantilever-based piezoelectric MEMS loudspeaker using static measurements and a lumped equivalent circuit in order to shorten the linearity optimization step of this type of loudspeakers in the design phase. Parameters are adapted to compare the results with the measurements of the Usound UT-P 2018, in order to validate the model.

## 2. Nonlinear State Space Model

**Figure 1:**
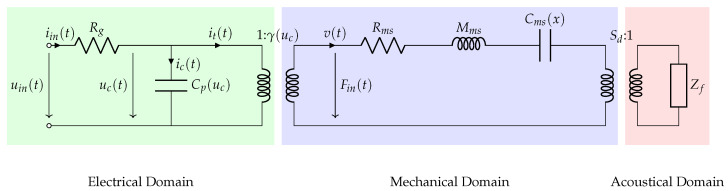
Equivalent electrical circuit of the loudspeaker.

As most loudspeakers behave as a simple mechanical oscillator for a wide range of frequencies, they can be well approximated as a first-order mechanical oscillator, two transformers and an electrical circuit. In the case of piezoelectric loudspeakers, the usual gyrator used for the electromechanical transduction is replaced by a transfomer, and the electrical circuit is a simple capacitor. The equivalent electrical circuit of the loudspeaker detailed in [32] is presented in Figure 1, where $R_{g}$ is the output resistance of the amplifier and the resistance of the connection lines, $C_{p}$ is the piezoelectric capacitance, γ is the transduction ratio of the piezoelectric layer, $R_{ms}$ represents the viscous losses of the mechanical oscillator, $M_{ms}$ is the moving mass of the loudspeaker, $C_{ms}$ is the compliance of the loudspeaker and $S_{d}$ is the effective radiating area of the membrane.

**Figure 2:**
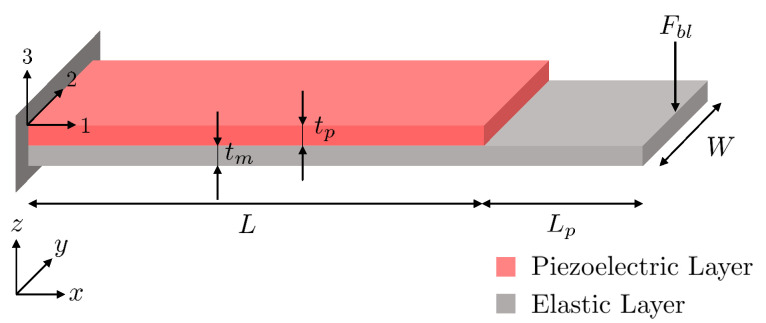
Schematic representation of a unimorph piezoelectric cantilever.

In the case of cantilever-based piezoelectric loudspeakers, the transduction factor γ can be estimated using the unimorph piezoelectric cantilever model described in [33]. A schematic representation of a unimorph cantilever is depicted in Figure 2 with the relevant dimensions and axes. Considering the cantilever clamped on one end and guided on the other end and using a null displacement at the tip of the cantilever, the blocked force $F_{bl}$ generated by the actuators as a function of the input voltage can be written:

(1) $$F_{bl}=\frac{6Wt_{p}}{4s_{11}^{p}L}\frac{AB(B+1)}{AB+1}\left(1-\frac{k_{p}}{k_{p}+k_{a}}\right)e_{31}\left(s_{11}^{p}+s_{12}^{p}\right)u_{c}$$

where *W* is the width of the actuator, $t_{p}$ is the thickness of the piezoelectric layer, $s_{11}^{p}$ and $s_{12}^{p}$ are the elastic compliance of the piezoelectric layer at constant electric field in the 1 and 2 direction, *L* is the length of the cantilever, $t_{m}$ is the thickness of the elastic layer, $A=s_{11}^{p}/s_{11}^{m}$, $B=t_{m}/t_{p}$, and $k_{a}$ and $k_{p}$ are the apparent stiffnesses of the active and passive parts of length *L* and $L_{p}$ of the actuators. The transduction factor γ is the the ratio of the blocked force $F_{bl}$ over the voltage $u_{c}$.

**Figure 3:**
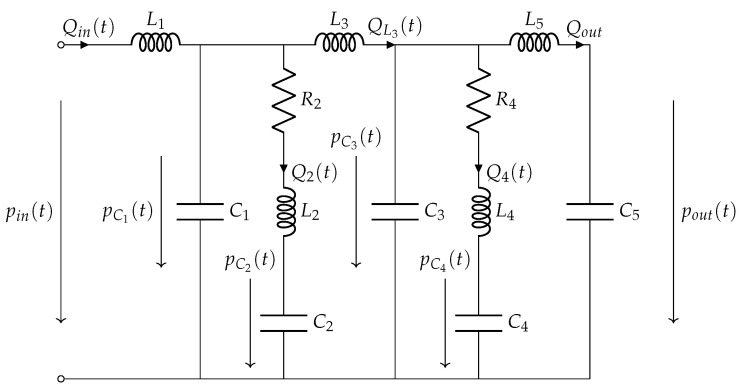
Lumped equivalent network of the coupler.

In our case, the load impedance $Z_{f}$ is the acoustical impedance seen by the front side of the membrane and is equal to the acoustical impedance of the coupler. Due to the size of diaphragm of MEMS loudspeakers, which are mostly smaller than 1 cm${}^{2}$, the back acoustic radiation impedance of the loudspeaker is not considered, due to its insignificant effect on the frequency response. A coupler is a mechanical device used to simulate the acoustical impedance of the average human ear, following the definition given in the IEC standard IEC 60318-4 [34]. The known electrical equivalent circuit is depicted in Figure 3.

Using the circuit resulting of the combination of Figure 1 and Figure 3, time domain differential equations can be derived. For the system to be fully defined, one state variable is needed for each capacitive or inductive element. The displacement *x* is also chosen, because it is used to evaluate the apparent stiffness of the actuators. The thirteen equations of the state variables are given in Appendix A. Equations (A1)–(A13) can be used in a matrix form and solved by using a differential approximation scheme [35]. The state space equation then writes:

(2) $$\frac{\partial }{\partial t}x=Ax+Bu_{in}\left(t\right)$$

with

(3) $$x=\left[\begin{array}{c} u_{c}\left(t\right)\\ v(t)\\ x(t)\\ F_{C_{ms}}\left(t\right)\\ p_{C_{1}}\left(t\right)\\ Q_{2}\left(t\right)\\ p_{C_{2}}\left(t\right)\\ Q_{L_{3}}\left(t\right)\\ p_{C_{3}}\left(t\right)\\ Q_{4}\left(t\right)\\ p_{C_{4}}\left(t\right)\\ Q_{out}\left(t\right)\\ p_{out}\left(t\right) \end{array}\right],$$

(4) $$A=\left[\begin{array}{ccccccccccccc} -\frac{1}{R_{g}C_{p}\left(u_{c}\right)} & -\frac{\gamma (u_{c})}{C_{p}\left(u_{c}\right)} & 0 & 0 & 0 & 0 & 0 & 0 & 0 & 0 & 0 & 0 & 0\\ \frac{\gamma (u_{c})}{M_{ms}+S_{d}^{2}L_{1}} & -\frac{R_{ms}}{M_{ms}+S_{d}^{2}L_{1}} & 0 & -\frac{1}{M_{ms}+S_{d}^{2}L_{1}} & -\frac{S_{d}}{M_{ms}+S_{d}^{2}L_{1}} & 0 & 0 & 0 & 0 & 0 & 0 & 0 & 0\\ 0 & 1 & 0 & 0 & 0 & 0 & 0 & 0 & 0 & 0 & 0 & 0 & 0\\ 0 & \frac{1}{C_{ms}\left(x\right)} & 0 & 0 & 0 & 0 & 0 & 0 & 0 & 0 & 0 & 0 & 0\\ 0 & \frac{S_{d}}{C_{1}} & 0 & 0 & 0 & -\frac{1}{C_{1}} & 0 & -\frac{1}{C_{1}} & 0 & 0 & 0 & 0 & 0\\ 0 & 0 & 0 & 0 & \frac{1}{L_{2}} & -\frac{R_{2}}{L_{2}} & -\frac{1}{L_{2}} & 0 & 0 & 0 & 0 & 0 & 0\\ 0 & 0 & 0 & 0 & 0 & \frac{1}{C_{2}} & 0 & 0 & 0 & 0 & 0 & 0 & 0\\ 0 & 0 & 0 & 0 & \frac{1}{L_{3}} & 0 & 0 & 0 & -\frac{1}{L_{3}} & 0 & 0 & 0 & 0\\ 0 & 0 & 0 & 0 & 0 & 0 & 0 & \frac{1}{C_{3}} & 0 & -\frac{1}{C_{3}} & 0 & -\frac{1}{C_{3}} & 0\\ 0 & 0 & 0 & 0 & 0 & 0 & 0 & 0 & \frac{1}{L_{4}} & -\frac{R_{4}}{L_{4}} & -\frac{1}{L_{4}} & 0 & 0\\ 0 & 0 & 0 & 0 & 0 & 0 & 0 & 0 & 0 & 0 & \frac{1}{C_{4}} & 0 & 0\\ 0 & 0 & 0 & 0 & 0 & 0 & 0 & 0 & \frac{1}{L_{5}} & 0 & 0 & 0 & -\frac{1}{L_{5}}\\ 0 & 0 & 0 & 0 & 0 & 0 & 0 & 0 & 0 & 0 & 0 & \frac{1}{C_{5}} & 0 \end{array}\right]$$

and

(5) $$B=\left[\begin{array}{c} \frac{1}{R_{g}C_{p}\left(u_{c}\right)}\\ 0\\ 0\\ 0\\ 0\\ 0\\ 0\\ 0\\ 0\\ 0\\ 0\\ 0\\ 0 \end{array}\right].$$

Equation (2) is of the form:

(6) $$\dot{x}=Ax+Bu$$

and thus, matrices can be discretized using a bilinear transform:

(7) $$A_{d}={\left(I-\frac{1}{2}AT_{s}\right)}^{-1}\left(I+\frac{1}{2}AT_{s}\right)$$

and

(8) $$B_{d}={\left(I-\frac{1}{2}AT_{s}\right)}^{-1}BT_{s}$$

with $T_{s}=1/f_{s}$ the sampling period. Using matrices $A_{d}$ and $B_{d}$ and an input signal, the output can be computed:

(9) $$x\left(k+1\right)=A_{d}x\left(k\right)+B_{d}u\left(k\right)$$

From the computed output pressure in the state vector x, the THD, which is the ratio between the sum of effective values of the harmonics and the effective value of the fundamental harmonic and other harmonics, is calculated using:

(10) $$\mathrm{THD}=100\cdot \frac{\sqrt{\sum_{h=2}^{H}v_{h}^{2}}}{\sqrt{\sum_{h=1}^{H}v_{h}^{2}}}$$

## 3. Static Measurements

As other loudspeakers state space models, this model uses nonlinear functions as input parameters. The nonlinear functions can be measured dynamically using adaptive state space models, calculated analytically, computed with finite element models or determined from static measurement. In this paper, we propose to use static measurements of the nonlinear transfer functions, measured on a commercial loudspeaker [36] and on PZT test devices. Since internal dimensions of the loudspeaker are unknown and difficult to measure, the lumped element parameters of the equivalent circuit $C_{ms}$, $S_{d}$, $M_{ms}$ and $C_{p}$ are taken directly from the data sheet.

### 3.1. Nonlinear Electromechanical Function

**Figure 4:**
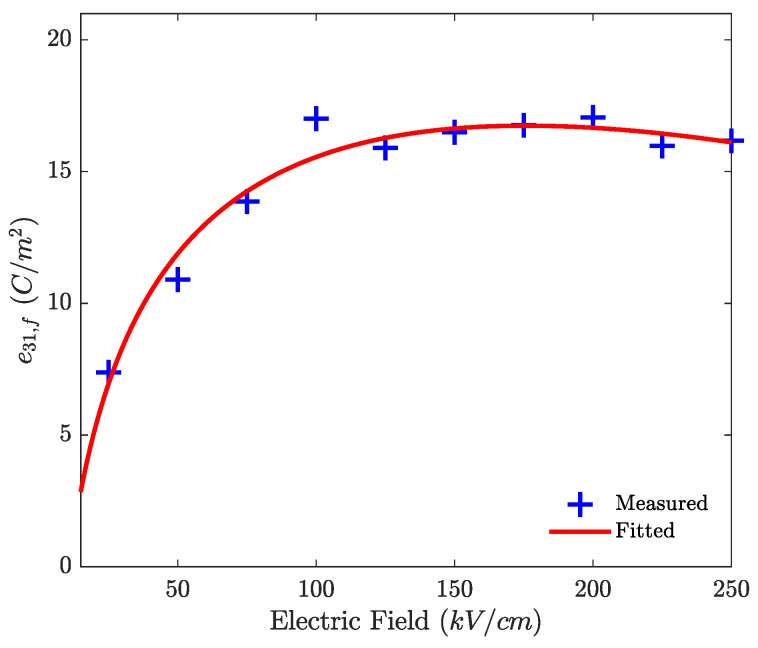
Measured and fitted function for the nonlinear transverse piezoelectric coefficient.

The $e_{31,f}$ piezoelectric coefficient of thin-film PZT is extracted from the $d_{33,f}$ measurement, with the procedure described in [37], as a function of the applied electric field, using the DBLI, on a standard PZT test device. As the piezoelectric coefficient $e_{31}$ is calculated for a piezoelectric material on wafer with this specific machine, it would be difficult to measure it on the loudspeaker directly. The parameter $e_{31}$ is known to be highly dependent on the applied electric field, due to intrinsic and extrinsic various effects, such as electrostriction, ferroelectric behavior and the domain movements. With the complete physical interactions being not completely understood, these nonlinearities are often simulated using phenomenological models, whose parameters are deduced from measurements [38,39]. The parameter $e_{31,f}$ and the fitted logarithmic function are depicted in Figure 4.

As it has been reported in multiple papers, the transversal piezoelectric coefficient of piezoelectric thin film increases for low electric fields, until it converges to the maximum value, due to the alignment of the domains of the material [40,41,42,43,44]. After the saturation of the rotation of the domains, the piezoelectric coefficient decreases slowly. The fitted function of $e_{31,f}$ is then used in Equation (1) to calculate the nonlinear transduction factor $\gamma (u_{c})$. Depending on the actuators geometry, the fitted $e_{31,f}$ function can be used in any other electromechanical transduction function.

### 3.2. Nonlinear Stiffness

**Figure 5:**
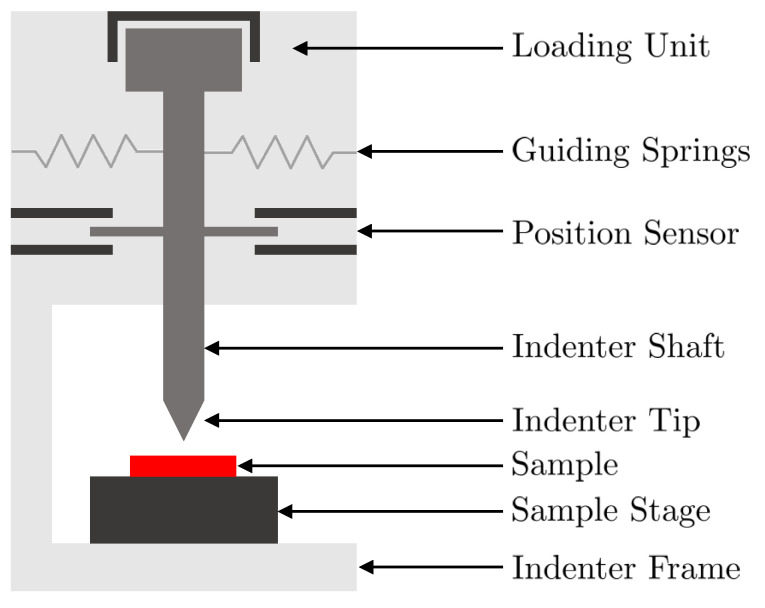
Schematic representation of a nanoindenter.

Using the setup depicted in Figure 5, the nonlinear relation between the force and the displacement of the commercial loudspeaker, not subjected to any voltage, is measured.

**Figure 6:**
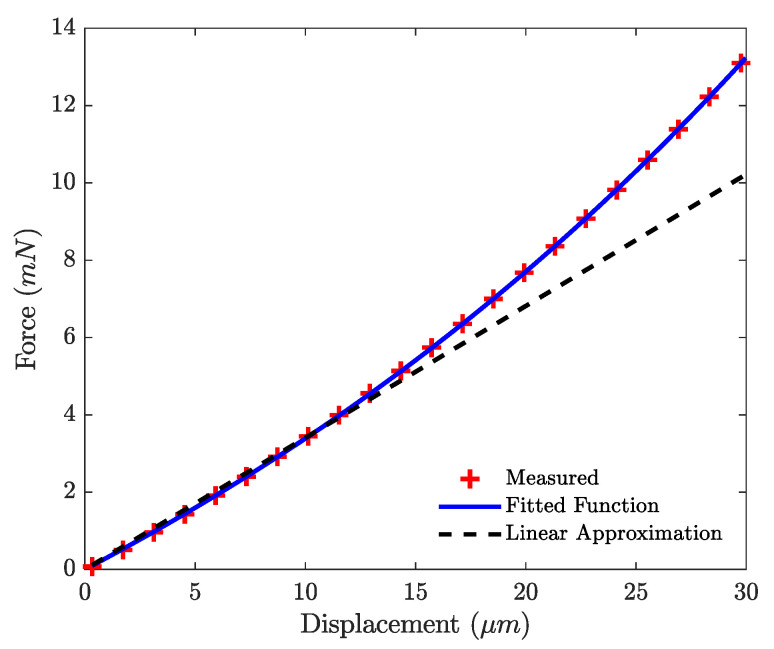
Nonlinear stiffness of the loudspeaker.

The commercial loudspeaker is placed on the sample loader. The nanoindenter, equipped with a spherical tip of radius 50 μm, applies a precise and well-known force on the moving part of the sample, until reaching the previously defined maximum displacement of 30 μm. After a processing of the raw data by the embedded software, the curve of the force applied on the sample and of the displacement of the shaft is retrieved and presented in Figure 6.

A third-order polynomial function, known to accurately represent the nonlinear behavior of clamped-guided structures, is used as the nonlinear fitting function. The linear approximation is calculated from the $V_{AS}$, which is the equivalent compliance volume value given in the loudspeaker data sheet, using:

(11) $$C_{ms}=\frac{V_{AS}}{\rho_{0}c_{0}^{2}S_{d}^{2}}$$

with $\rho_{0}$ the density of air at room temperature, $c_{0}$ the speed of sound in air at room temperature and $S_{d}$ the effective radiating surface of the loudspeaker [45]. From the fitted function, the nonlinear compliance $C_{ms}\left(x\right)$ can be calculated. This nonlinear function can also be calculated knowing the geometry of the loudspeaker and a finite element model or analytically.

### 3.3. Capacitance

**Figure 7:**
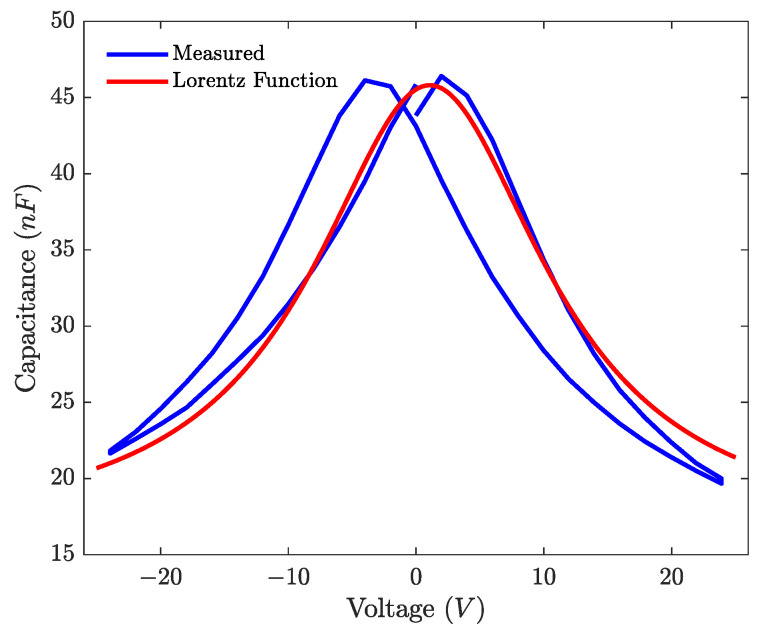
Non linear capacitance of the loudspeaker.

As the PZT thin film is ferroelectric, the capacitance created by the piezoelectric layer between the electrodes varies with the applied electric field. The nonlinear relation was measured on a layer of PZT using the AixACCT TF Analyzer 2000. The measured variation of the capacitance and the fitted Lorentz function are presented in Figure 7.

From the fitted Lorentzian function, the nonlinear capacitance $C_{p}\left(u_{c}\right)$ can be calculated. The curves can be adjusted in order to match the value of the data sheet of the loudspeaker.

## 4. Results

**Figure 8:**
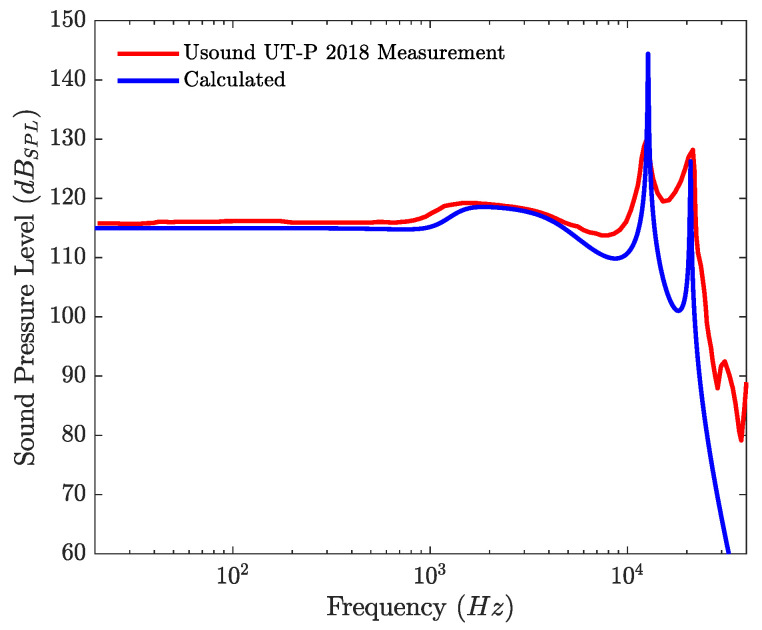
Simulated frequency response of the commercial loudspeaker and measured frequency response of the loudspeaker adapted from [36].

Using Equation (9) and a scaled dirac whose amplitude at the first sample is $f_{s}u_{p}/2$ as an input signal, the linear frequency response of the loudspeaker inside the coupler is computed. The computed frequency response is compared to the frequency response of the commercial loudspeaker, in Figure 8. The parameters for the circuits presented on Figure 1 and Figure 3 are derived or directly taken from the data sheet of the loudspeaker [36] and taken from the literature for the coupler [46]. Although the influence of $R_{g}$ is negligible, it is set to 1mΩ to have a properly scaled $A_{d}$ matrix. The value of $M_{ms}$ is computed from the previously determined value of $C_{ms}$ and the value of the resonance frequency given in the data sheet using:

(12) $$M_{ms}=\frac{1}{4\pi^{2}f_{s}^{2}C_{ms}}$$

The value of $R_{ms}$ is determined using the quality factor of the data sheet using:

(13) $$R_{ms}=\frac{1}{Q}\sqrt{\frac{M_{ms}}{C_{ms}}}$$

All parameters are summarized in Table 1.

Despite some high-frequency mismatches, due to the modal behavior of the membrane above 20 kHz, and a slight difference between the quality factors of the coupler resonances at, respectively, 12,710 and 20,970 Hz, the simulated frequency response gives a good approximation of the manufacturer measurement.

**Figure 9:**
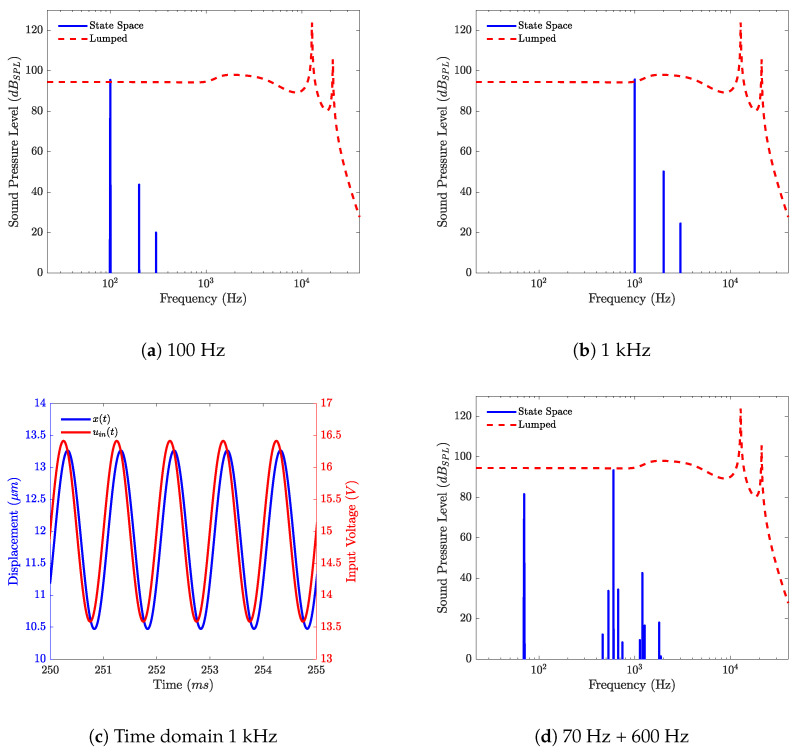
Nonlinear loudspeaker model responses to different input signals with the linear frequency response as a reference in frequency and time domain.

Using the identified nonlinear functions for parameters γ, $K_{ms}$ and $C_{p}$ and a pure sine of 1 kHz of amplitude 1.41 V as an input signal, the Fourier transform of the output signal can be calculated and is presented in Figure 9, for different input signals, as well as the linear frequency response.

In Figure 9a,b, the response of the loudspeaker to a pure sine of, respectively, 100 Hz and 1 kHz are given, and one can see the second and third harmonic clearly appear. In Figure 9c, the displacement and input voltage in time domain are given. In Figure 9d, the response of the loudspeaker to a sum of two pure sines with $f_{1}=$ 70 Hz and $f_{2}$ = 600 Hz, is depicted, and one can see that the intermodulation appears with the components of frequencies $f_{2}\pm f_{1}$, $f_{2}\pm 2f_{1}$, $2f_{2}\pm f_{1}$ and $3f_{2}\pm f_{1}$.

**Figure 10:**
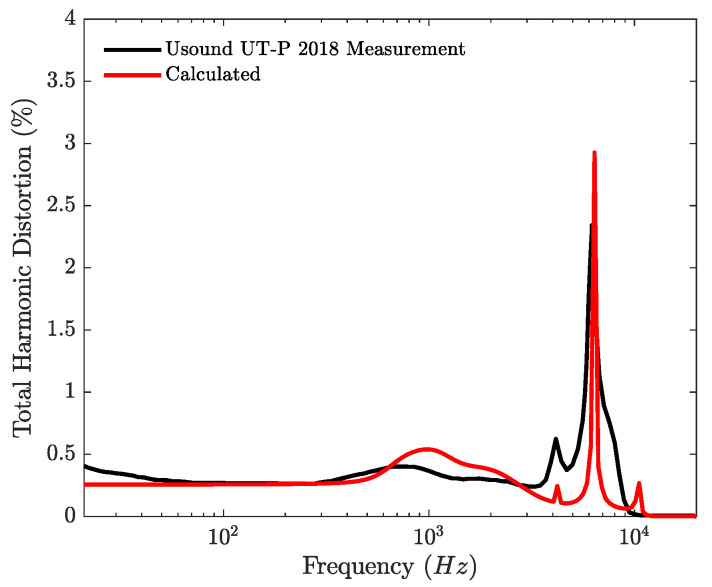
Simulated THD of the commercial loudspeaker and measured THD of the loudspeaker adapted from [36].

The THD is computed point by point using a pure sine as the input signal for 200 points. The simulated THD is compared to the THD measured by the manufacturer in Figure 10. The pressure signal is low pass filtered at 16 kHz before the THD estimation, as it is described in the data sheet of the loudspeaker. There is a good agreement between the absolute level of the THD between 40 and 200 Hz, where the difference between the two curves is below 20%. Above 500 Hz, the difference between the simulated and measured frequency responses is the cause of the difference between the THD curves and around the distortion peak at about 6.4 kHz, the difference is maximum and above 100%. Qualitatively, the shape of the curve around the resonance of the loudspeaker has a similar shape, capturing the effect of the resonance of the loudspeaker and the one of the coupler on the THD.

**Figure 11:**
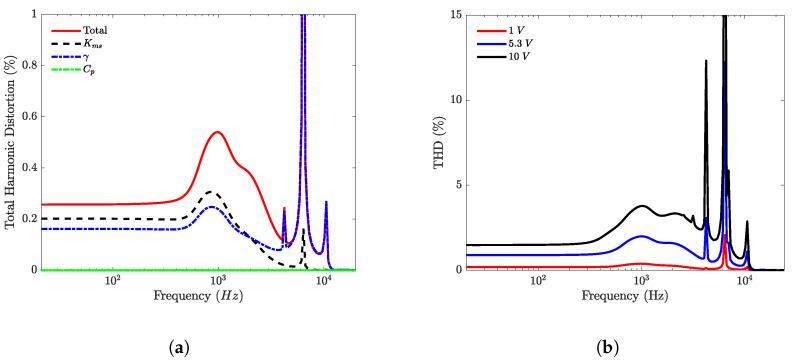
THD generated by individual parameters (**a**) and THD as a function of input level (**b**).

In Figure 11a, the respective contributions of $\gamma (u_{c})$, $C_{ms}\left(x\right)$ and $C_{p}\left(u_{c}\right)$ to the total harmonic distortion are shown. As the displacement, the harmonic distortion generated by $C_{ms}\left(x\right)$ is maximum below the resonance frequency of the loudspeaker and negligible above the resonance frequency. The harmonic distortion generated by the transduction factor $\gamma (u_{c})$ is modulated by the frequency response of the loudspeaker but remains high in the whole audio bandwidth. The contribution of $C_{p}\left(u_{c}\right)$ is three orders of magnitude below the other ones and then negligible. In Figure 11b, the THD for three different input levels is presented.

## 5. Conclusions

The model presented in this paper allows one to evaluate the THD generated by a cantilever-based MEMS loudspeaker, by only measuring the nonlinear relations between the stiffness and the displacement, the capacitance and the electric field, and the force and the electric field. It also could allow one to use predistortion techniques to reduce the said total harmonic distortion in MEMS loudspeakers. Although open-loop correction of loudspeaker nonlinearities is tedious for non-MEMS loudspeakers, due to large manufacturing discrepancies, this technique could be more suited for MEMS design, due to the tightest tolerances of the manufacturing processes. This model is also very efficient in terms of computational power compared to a finite element modeling solution and could be used in the early stages of piezoelectric MEMS loudspeakers development, to evaluate the linear performance of the loudspeaker. The discrepancies between the model and the measurement can be attributed to the assumptions made in the model of the loudspeaker. For example, the nonlinear behavior of the PZT is known to widely vary with frequency and to create hysteresis. In addition, other different nonlinear parameters were not taken into account, such as the nonlinearities due to high velocity in the coupler slits, but can be added easily to the model.

## Figures and Tables

**Table 1:** Table of lumped elements circuits parameters.

Parameter	Value	Unit
$R_{g}$	1	mΩ
$C_{p}$	39	nF
$R_{ms}$	$5\times 10^{-3}$	Ns/m
$M_{ms}$	1.16	mg
$C_{ms}$	$3\times 10^{-3}$	m/N
$S_{d}$	12	mm${}^{2}$
$L_{1}$	82.9	Pas${}^{2}$/m${}^{3}$
$L_{2}$	9400	Pas${}^{2}$/m${}^{3}$
$L_{3}$	130.3	Pas${}^{2}$/m${}^{3}$
$L_{4}$	983.8	Pas${}^{2}$/m${}^{3}$
$L_{5}$	133.4	Pas${}^{2}$/m${}^{3}$
$C_{1}$	$1\times 10^{-12}$	Pa/m${}^{3}$
$C_{2}$	$1.9\times 10^{-12}$	Pa/m${}^{3}$
$C_{3}$	$1.5\times 10^{-12}$	Pa/m${}^{3}$
$C_{4}$	$2.1\times 10^{-12}$	Pa/m${}^{3}$
$C_{5}$	$1.517\times 10^{-12}$	Pa/m${}^{3}$
$R_{1}$	$50.6\times 10^{6}$	Pas/m${}^{3}$
$R_{2}$	$31.1\times 10^{6}$	Pas/m${}^{3}$

## Abbreviations

The following abbreviations are used in this manuscript:

THD	Total Harmonic Distortion
MEMS	Micro Electromechanical Systems
IEC	International Electrotechnical Commission
DUT	Device Under Test
DBLI	Double-Beam Laser Interferometer
PZT	Lead Zirconate Titanate

## Appendix A. State Space Equations

(A1)
$$\frac{\partial u_{c}\left(t\right)}{\partial t}=\frac{1}{R_{g}C_{p}\left(u_{c}\right)}u_{in}\left(t\right)-\frac{1}{R_{g}C_{p}\left(u_{c}\right)}u_{c}\left(t\right)-\frac{\gamma (u_{c})}{C_{p}\left(u_{c}\right)}v\left(t\right)$$

(A2)
$$\begin{array}{cc} \frac{\partial v(t)}{\partial t}= & \frac{\gamma (u_{c})}{M_{ms}+S_{d}^{2}L_{1}}u_{c}\left(t\right)-\frac{R_{ms}}{M_{ms}+S_{d}^{2}L_{1}}v\left(t\right)\\ \; & \;-\frac{1}{M_{ms}+S_{d}^{2}L_{1}}F_{C_{ms}}\left(t\right)-\frac{S_{d}}{M_{ms}+S_{d}^{2}L_{1}}p_{C_{1}}\left(t\right) \end{array}$$

(A3)
$$\frac{\partial F_{C_{ms}}}{\partial t}=\frac{1}{C_{ms}\left(x\right)}v\left(t\right),$$

(A4)
$$\frac{\partial x(t)}{\partial t}=v\left(t\right),$$

(A5)
$$\frac{\partial p_{C_{1}}\left(t\right)}{\partial t}=\frac{S_{d}}{C_{1}}v\left(t\right)-\frac{1}{C_{1}}Q_{2}\left(t\right)-\frac{1}{C_{1}}Q_{L_{3}}\left(t\right)$$

(A6)
$$\frac{\partial Q_{2}\left(t\right)}{\partial t}=\frac{1}{L_{2}}p_{C_{1}}\left(t\right)-\frac{1}{L_{2}}p_{C_{2}}\left(t\right)-\frac{R_{2}}{L_{2}}Q_{2}\left(t\right)$$

(A7)
$$\frac{\partial p_{C_{2}}\left(t\right)}{\partial t}=\frac{1}{C_{2}}Q_{2}\left(t\right)$$

(A8)
$$\frac{\partial Q_{L_{3}}\left(t\right)}{\partial t}=\frac{1}{L_{3}}p_{C_{1}}\left(t\right)-\frac{1}{L_{3}}p_{C_{3}}\left(t\right)$$

(A9)
$$\frac{\partial p_{C_{3}}\left(t\right)}{\partial t}=\frac{1}{C_{3}}Q_{L_{3}}\left(t\right)-\frac{1}{C_{3}}Q_{4}\left(t\right)-\frac{1}{C_{3}}Q_{out}\left(t\right)$$

(A10)
$$\frac{\partial Q_{4}\left(t\right)}{\partial t}=\frac{1}{L_{4}}p_{C_{3}}\left(t\right)-\frac{1}{L_{4}}Q_{4}\left(t\right)-\frac{1}{L_{4}}p_{C_{4}}\left(t\right)$$

(A11)
$$\frac{\partial p_{C_{4}}\left(t\right)}{\partial t}=\frac{1}{C_{4}}Q_{4}\left(t\right)$$

(A12)
$$\frac{\partial Q_{out}\left(t\right)}{\partial t}=\frac{1}{L_{5}}p_{C_{3}}\left(t\right)-\frac{1}{L_{5}}p_{out}\left(t\right)$$

(A13)
$$\frac{\partial p_{out}\left(t\right)}{\partial t}=\frac{1}{C_{5}}Q_{out}\left(t\right)$$
